# Addressing the U.S. maternal health crisis: a systematic review of healthcare access barriers, disparate outcomes, and effective interventions

**DOI:** 10.3389/fpubh.2026.1814063

**Published:** 2026-04-23

**Authors:** Mohammad Najeh Samara, Kimberly Harry, Aish Barua

**Affiliations:** School of Systems Science and Industrial Engineering, Binghamton University, Binghamton, NY, United States

**Keywords:** cultural sensitivity, healthcare access, healthcare equity, healthcare interventions, maternal health disparities, maternal mortality, racial disparities, severe maternal morbidity

## Abstract

**Background:**

The United States faces a profound maternal health crisis despite its global economic standing, with maternal mortality rates significantly higher than other high-income countries and stark disparities across racial and ethnic lines. This systematic literature review aims to synthesize current evidence on barriers to maternal healthcare access, resulting health outcomes, and effective interventions for reducing disparities.

**Methods:**

Following PRISMA guidelines, a comprehensive search was conducted across PubMed, Web of Science, and ProQuest for literature published January 2000 and December 2024. The initial search yielded 122,756 records, which were narrowed to 44 studies for final synthesis after applying inclusion/exclusion criteria. Thematic analysis identified patterns related to barriers, outcomes, and interventions in maternal healthcare.

**Results:**

The systematic search identified 44 studies for synthesis. Analysis revealed five major barrier categories affecting maternal healthcare access: financial (24 references), systematic (34 references), educational (6 references), geographical (17 references), and comorbidity-related (1 reference). These barriers contributed to concerning disparities in maternal health outcomes, with Black women experiencing mortality rates 3-4 times higher than White women. Promising interventions included safety net providers (such as community health centers, mental health teams), culturally sensitive approaches, healthcare workers (doulas, translators), predictive models, special initiatives, and policy changes (Medicaid expansion, telehealth).

**Conclusion:**

Addressing the U.S. maternal health crisis requires coordinated action targeting multifaceted barriers and implementing evidence-based interventions. Recommendations include expanding Medicaid coverage beyond the current postpartum limit, implementing approaches addressing social determinants of health, integrating diverse healthcare workers into maternal care teams, and prioritizing culturally sensitive, patient-informed care while actively addressing systematic barriers, particularly racism and discrimination.

## 1 Introduction

### 1.1 Background

The United States faces a profound maternal health crisis that stands in stark contrast to its global economic standing. Despite being one of the wealthiest nations, the U.S. reports the highest maternal mortality rate among high-income countries, with rates more than 10 times higher than some peers (1, 2). Maternal mortality surveillance in the United States faces significant methodological challenges, with the World Health Organization employing seven different estimation methods (3). National Vital Statistics System data shows U.S. rates of 17.4 per 100,000 in 2018 compared to 1.7 in New Zealand and 6.5 in the United Kingdom (4). However, pregnancy checkbox misclassification may inflate U.S. figures (5, 6), with alternative methods suggesting more stable rates (7). Critically, racial disparities persist across all methodologies. Geographic variations further underscore the crisis, as predominantly rural states such as Alabama, Arkansas, and Mississippi report maternal mortality rates exceeding 40 per 100,000 live births (8), reflecting limited local access to obstetric care, long travel distances, and the widespread closure of rural hospitals over the past decade (9). Most concerning is the disproportionate impact on racial and ethnic minorities. Black women experience maternal mortality rates 3 to 4 times higher than their White counterparts (10), with African American women in the United States, facing rates reaching 69.9 per 100,000 live births (2). Even when controlling for education, income, and access to prenatal care, these disparities persist (10). Indigenous women face even worse outcomes, highlighting the profound impact of systemic racism and colonialism (11). These disparities are inextricably linked to systemic inequities in healthcare access, compounded by socioeconomic and geographic factors (1, 12).

Multiple interconnected factors contribute to this crisis. Clinically, inconsistent obstetric practices and lack of standardized emergency protocols compromise care quality (13). Cardiovascular diseases have emerged as a leading cause of maternal mortality, with significant increases in deaths related to cardiomyopathy (1, 14). The prevalence of chronic conditions such as obesity, diabetes, and hypertension has risen, complicating pregnancies and increasing adverse outcome risks (15, 16). Age-related risks have become more prominent, with women aged 35 and older facing higher mortality risks from conditions like obstetric hemorrhage and postpartum cardiomyopathy (17). Mental health issues and substance use disorders represent significant but under-recognized contributors to maternal deaths (18), while unintentional deaths from violence, overdose, and self-harm are emerging contributors (12).

Addressing this complex, multifactorial crisis requires comprehensive understanding of the barriers, outcomes, and interventions that shape maternal healthcare across diverse populations and regions. While individual studies have examined specific aspects of maternal health disparities, a systematic synthesis examining the full spectrum of barriers, outcomes, and evidence-based interventions is needed to inform coordinated action. This systematic literature review aims to fill this gap by synthesizing current evidence, critically evaluating existing efforts, and identifying opportunities for more equitable maternal health outcomes.

This review is guided by three fundamental research questions:

RQ1: Among women of reproductive age in the United States (P), what financial, geographic, systemic, educational, and comorbidity-related barriers (exposure) are associated with reduced access to maternal healthcare (O), as reported in empirical quantitative, qualitative, and mixed-methods studies (S)?

RQ2: Among racially, ethnically, and socioeconomically diverse populations of pregnant and postpartum women in the United States (P), how do clinical, social, and structural determinants (exposure) contribute to disparities in maternal mortality and severe maternal morbidity (O), compared across demographic subgroups including race/ethnicity, income, and geography (C), based on empirical evidence published between 2000 and 2024 (S)?

RQ3: Among women of reproductive age in the United States, particularly those from underserved or high-disparity populations (P), what clinical, community-based, and policy-level interventions (I) have been implemented or evaluated to improve equitable access to maternal healthcare and reduce adverse maternal outcomes (O), compared to standard or no intervention where reported (C), across empirical studies (S)?

These questions were structured in accordance with the PICOS framework (Population, Intervention/Exposure, Comparison, Outcome, Study design) to ensure systematic alignment between the review questions, eligibility criteria, and synthesis approach (19).

## 2 Methodology

### 2.1 Research design

**Figure 1:**
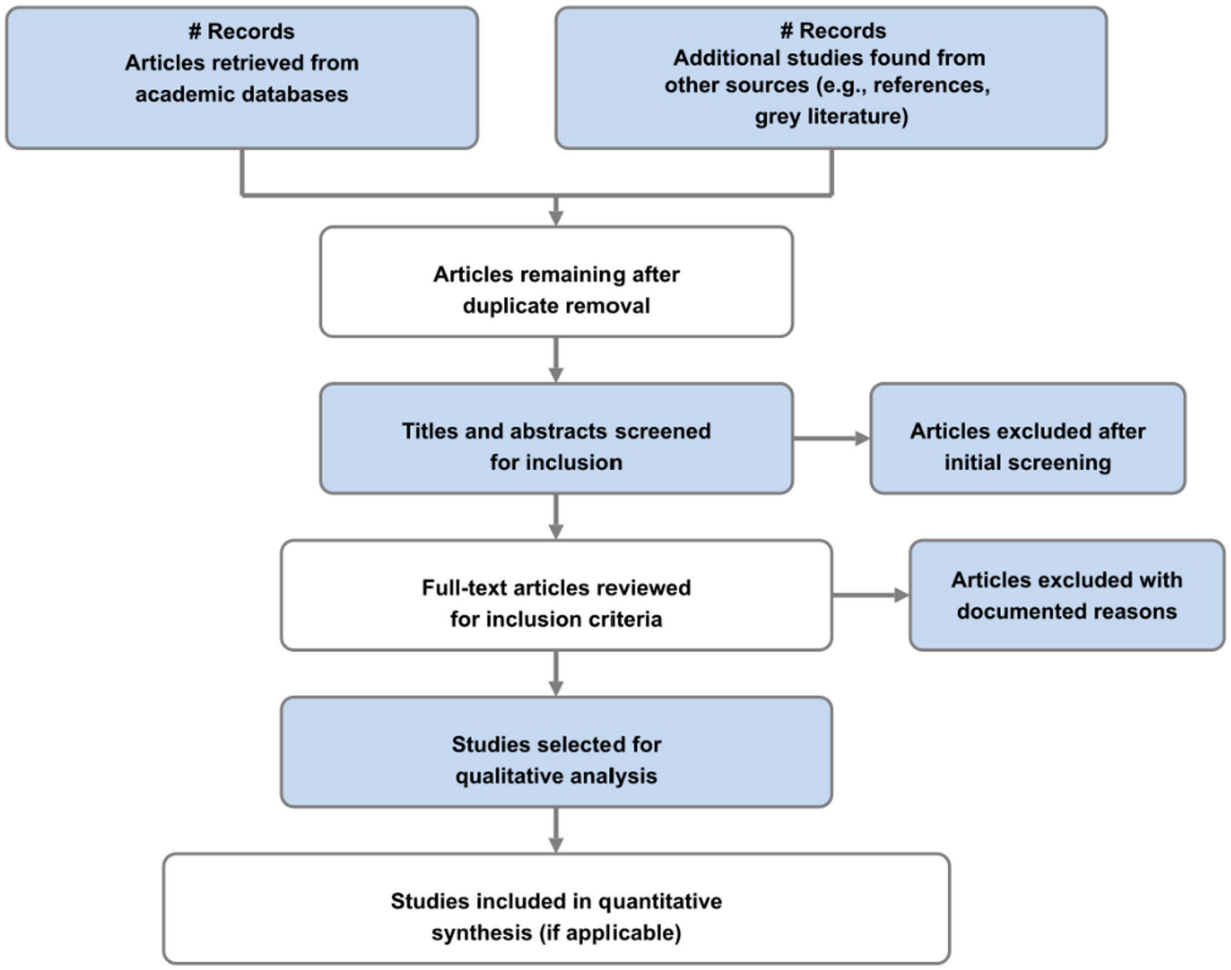
Study selection flowchart following PRISMA guidelines.

This systematic literature review followed PRISMA (Preferred Reporting Items for Systematic Reviews and Meta-Analyses) guidelines (illustrated in Figure 1) and utilized the PRISMA checklist to ensure transparency, reproducibility, and methodological rigor (20). The review incorporated established recommendations for conducting qualitative systematic reviews in health research (21, 22), employing a mixed-methods synthesis approach that integrated both quantitative findings on maternal mortality and morbidity rates with qualitative insights on barriers to care and patient experiences [“Synthesizing the Evidence,” (23)]. An equity lens was applied throughout the entire process—from search strategy development to data synthesis—to ensure meaningful attention to socioeconomic, racial, ethnic, and geographic disparities in maternal healthcare access and outcomes (24).

### 2.2 Search strategy

A comprehensive search strategy was developed to identify relevant literature across multiple academic databases. Three primary databases were selected for their coverage of medical, social science, and interdisciplinary research: PubMed, Web of Science, and ProQuest. The search covered literature published between January 2000 and December 2024 to capture both historical trends and contemporary developments in maternal healthcare access and outcomes. The search strategy employed specific terms organized into three conceptual groups connected by Boolean operators, as outlined in Table 1.

**Table 1:** Search strategy components used in the systematic review.

Search strategy component	Description
Search Terms Group 1	matern* N5 health OR matern* N5 healthcare OR matern* N5 care OR prenatal health OR prenatal healthcare OR perinatal health OR perinatal healthcare
Search Terms Group 2	death OR fatal* OR mortality OR morbidity
Search Terms Group 3	equitable access OR equi* access OR fair access OR inclusive access OR disparit* OR SDOH OR “social determinants of health” OR increase access
Boolean Operator	AND (connecting the three groups)
Publication Type	Scholarly work: journal articles, conference proceedings, theses/dissertations

The proximity operator (N5) was utilized to ensure that maternal-related terms appeared within five words of healthcare-related terms, thereby enhancing the precision of the search and focusing on literature specifically addressing maternal healthcare concerns.

Table 2 presents the complete, database-specific search strings applied in each repository, including all Boolean operators, proximity operators, and field tags used to ensure reproducibility.

**Table 2:** Database-specific search strings used in the systematic review.

Database	Full search string
PubMed	(“maternal health”[tiab] OR “maternal healthcare”[tiab] OR “maternal care”[tiab] OR “prenatal health”[tiab] OR “prenatal healthcare”[tiab] OR “perinatal health”[tiab] OR “perinatal healthcare”[tiab] OR “Maternal Health”[MeSH] OR “Prenatal Care”[MeSH] OR “Perinatal Care”[MeSH]) AND (“death”[tiab] OR “mortality”[tiab] OR “morbidity”[tiab] OR “Maternal Mortality”[MeSH] OR “Maternal Morbidity”[MeSH]) AND (“equitable access”[tiab] OR “health disparities”[tiab] OR “disparities”[tiab] OR “SDOH”[tiab] OR “social determinants of health”[tiab] OR “Healthcare Disparities”[MeSH] OR “Social Determinants of Health”[MeSH] OR “Health Equity”[MeSH]) AND (“United States”[MeSH] OR “United States”[tiab]) AND (“2000/01/01”[PDAT]: “2024/12/31”[PDAT])
Web of Science	TS = ((matern* NEAR/5 health) OR (matern* NEAR/5 healthcare) OR (matern* NEAR/5 care) OR “prenatal health” OR “prenatal healthcare” OR “perinatal health” OR “perinatal healthcare”) AND TS = (death OR fatal* OR mortality OR morbidity) AND TS = (“equitable access” OR (equi* NEAR/3 access) OR “fair access” OR “inclusive access” OR disparit* OR SDOH OR “social determinants of health” OR “increase access”) AND CU = (United States) AND PY = (2000-2024) AND LA = (English) AND DT = (Article OR Proceedings Paper OR Dissertation)
ProQuest	(matern* N5 health OR matern* N5 healthcare OR matern* N5 care OR “prenatal health” OR “prenatal healthcare” OR “perinatal health” OR “perinatal healthcare”) AND (death OR fatal* OR mortality OR morbidity) AND (“equitable access” OR equi* N3 access OR “fair access” OR “inclusive access” OR disparit* OR SDOH OR “social determinants of health” OR “increase access”) AND (“United States”) AND pd.(20000101-20241231) AND la(English) AND dtype(journal article OR conference proceeding OR dissertation)

[tiab], title/abstract field tag; [MeSH], Medical Subject Headings; NEAR/5 = within five words (Web of Science); N5 = within five words (ProQuest); TS = topic field; CU = country; PY = publication year; pd = publication date; la = language; dtype = document type.

### 2.3 Eligibility criteria

To ensure relevance and methodological rigor, studies were selected based on predefined inclusion and exclusion criteria, as outlined in Table 3.

**Table 3:** Inclusion and exclusion criteria for study selection.

Criteria type	Details
Inclusion criteria	1- Published in English
	2- Focused on maternal healthcare in the United States
	3- Empirical studies using quantitative, qualitative, or mixed methods
	4- Addressed healthcare accessibility, maternal outcomes, or interventions
	5- Published between January 2000 and December 2024
Exclusion criteria	1- Conducted outside the United States or focused on international contexts
	2- Non-empirical research (e.g., opinion pieces, editorials, commentaries)
	3- Not focused specifically on maternal healthcare
	4- Did not address issues related to equity, accessibility, or maternal outcomes

### 2.4 Quality assessment

Two independent reviewers conducted quality assessments during full-text screening using the Mixed Methods Appraisal Tool (25), which was selected for its applicability across quantitative, qualitative, and mixed-methods study designs consistent with the scope of this review. Studies were evaluated based on relevance to the research questions, empirical robustness, and clarity of reporting. Priority was given to studies with transparent methodological descriptions, explicit attention to equity and access, and overall methodological soundness. Disagreements were resolved through discussion and consensus. Studies that failed to meet minimum quality criteria were excluded during full-text review. For included studies, risk of bias was assessed using MMAT design-specific criteria addressing sampling adequacy, measurement validity, and potential for confounding (25). Studies with higher risk of bias were retained but weighted accordingly during synthesis, with quality-related limitations acknowledged in the Discussion where relevant.

### 2.5 Data extraction, coding, and analysis

Following final selection, a standardized data extraction form was applied independently by both reviewers to each included study prior to consensus reconciliation. The form captured author(s) and year of publication, study design, sample characteristics, key outcomes reported, and primary findings relevant to the three research questions (summarized in Supplementary Table 1). Discrepancies between reviewers were resolved through discussion until consensus was reached. The extracted evidence was subsequently imported into NVivo qualitative analysis software for thematic coding. For barriers, codes included financial constraints, insurance limitations, systemic issues, education, geography, and comorbidities. For outcomes, themes included morbidity, mortality, mental health, and birth complications. Intervention codes highlighted approaches such as culturally sensitive care, [defined as language-concordant services, incorporation of cultural practices, and demographically representative care teams (26)], Medicaid expansion, community health initiatives, and telehealth (noting that 22%–50% of rural populations lack adequate broadband infrastructure, limiting telehealth accessibility in areas with greatest maternal health disparities) (27) This coding process enabled synthesis of both quantitative trends (e.g., statistical reports of mortality rates) and qualitative insights (e.g., descriptions of barriers and patient experiences).

### 2.6 Synthesis of findings

An integrative approach combined quantitative and qualitative evidence to answer research questions holistically. Quantitative data provided overview of trends, such as elevated mortality rates among Black and Hispanic women and prevalence of comorbidities. Qualitative data offered deeper insight into contextual factors, including patient experiences with discrimination, financial hardship, and geographic isolation. Findings related to interventions were similarly integrated, drawing on descriptive and evaluative studies to assess the impact of various strategies drawing on descriptive and evaluative studies to assess the impact of various strategies, including coverage- and access-oriented approaches whose effectiveness is shaped by broader structural constraints within the healthcare financing system. By merging different types of evidence, the synthesis captured the complex and multifaceted nature of maternal health disparities in the United States.

## 3 Results

**Figure 2:**
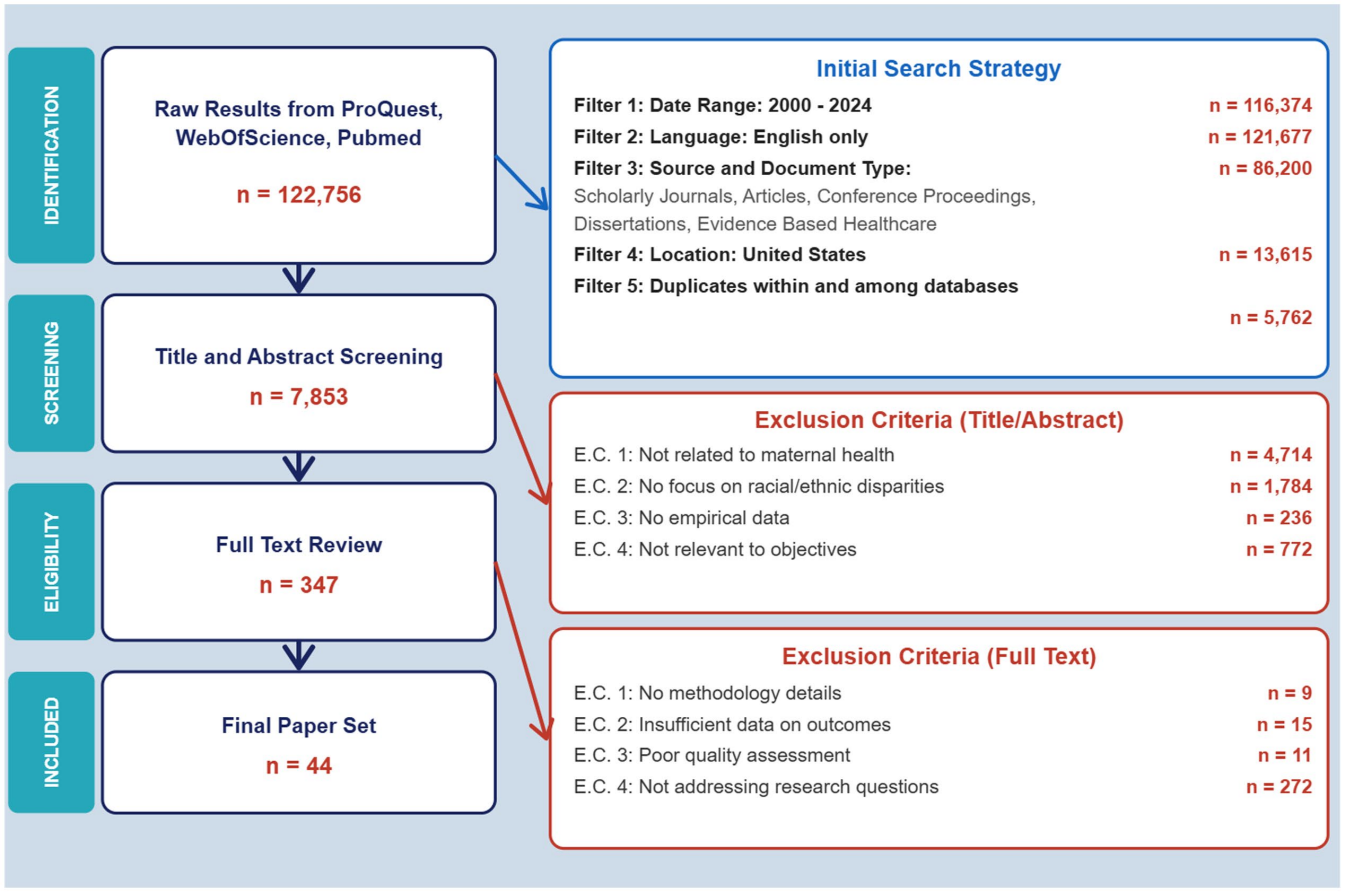
Flow diagram of study selection process.

A multi-stage screening process was conducted to identify eligible studies. The initial search across ProQuest, Web of Science, and PubMed yielded 122,756 records, which were reduced to 86,200 after applying date, language, and publication-type filters. Limiting studies to those conducted in the United States and removing duplicates resulted in 7,853 records screened by title and abstract. Of these, 347 articles underwent full-text review, and 44 studies met all inclusion criteria and were included in the final synthesis. The study selection process is summarized in Figure 2.

### 3.1 What barriers exist in accessing maternal healthcare?

**Figure 3:**
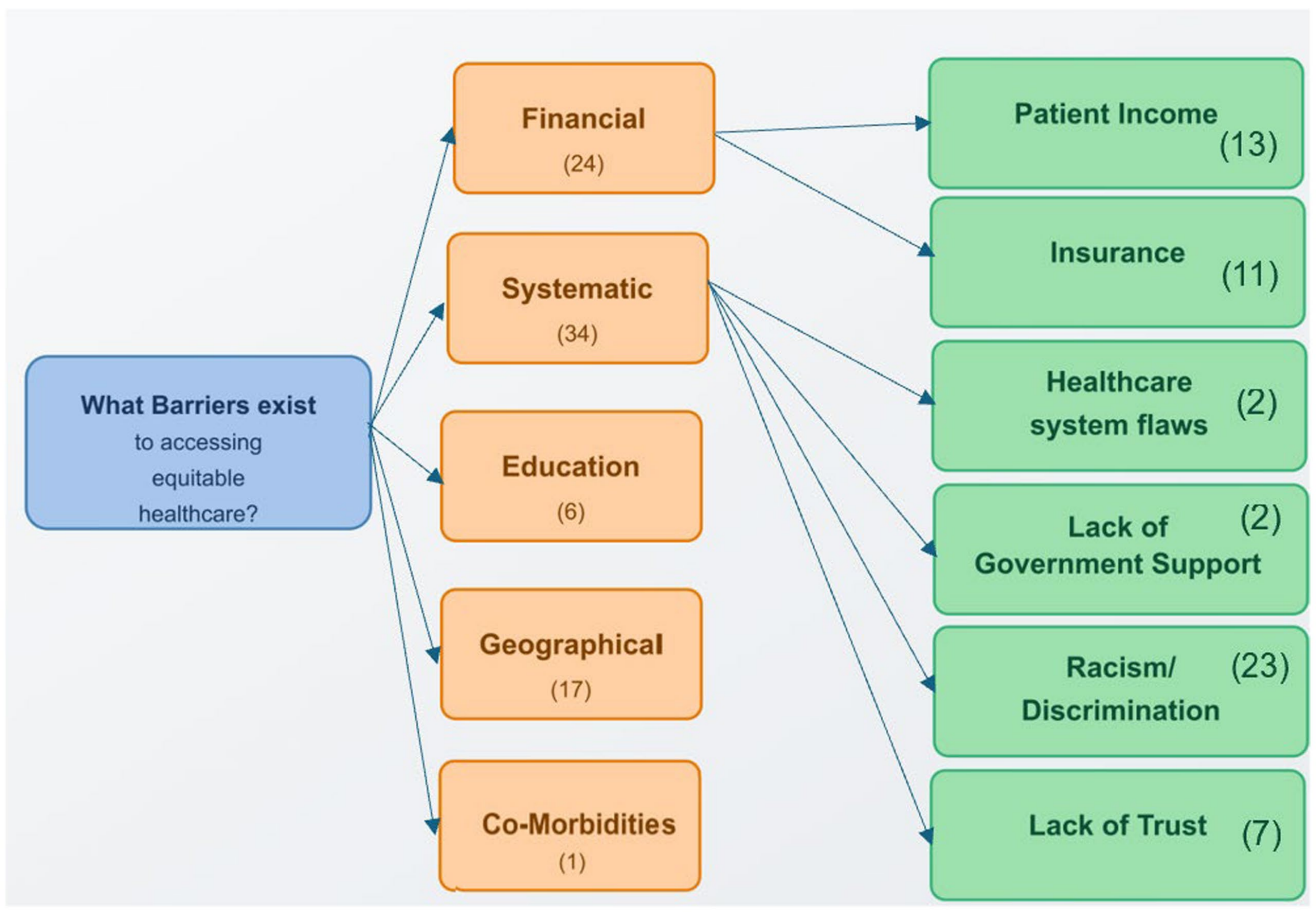
Categorization of barriers to equitable maternal healthcare access identified in the systematic review. Numbers in parentheses indicate the frequency of references to each barrier category across the systematic review. References to different barrier types within the same publication were counted separately to enable a more detailed analysis of the various obstacles to maternal healthcare access. This methodological approach allows for a more nuanced understanding of how multiple barriers often intersect to create compounded challenges for vulnerable populations.

The review identified five major categories of barriers to equitable maternal healthcare access: financial, systematic, educational, geographical, and comorbidity-related. These barriers reflect intersecting challenges related to resources, healthcare systems, education, location, and pre-existing health conditions that contribute to disparities in access and outcomes. Figure 3 summarizes these barrier categories and their subcomponents.

#### 3.1.1 Financial barriers

**Figure 4:**
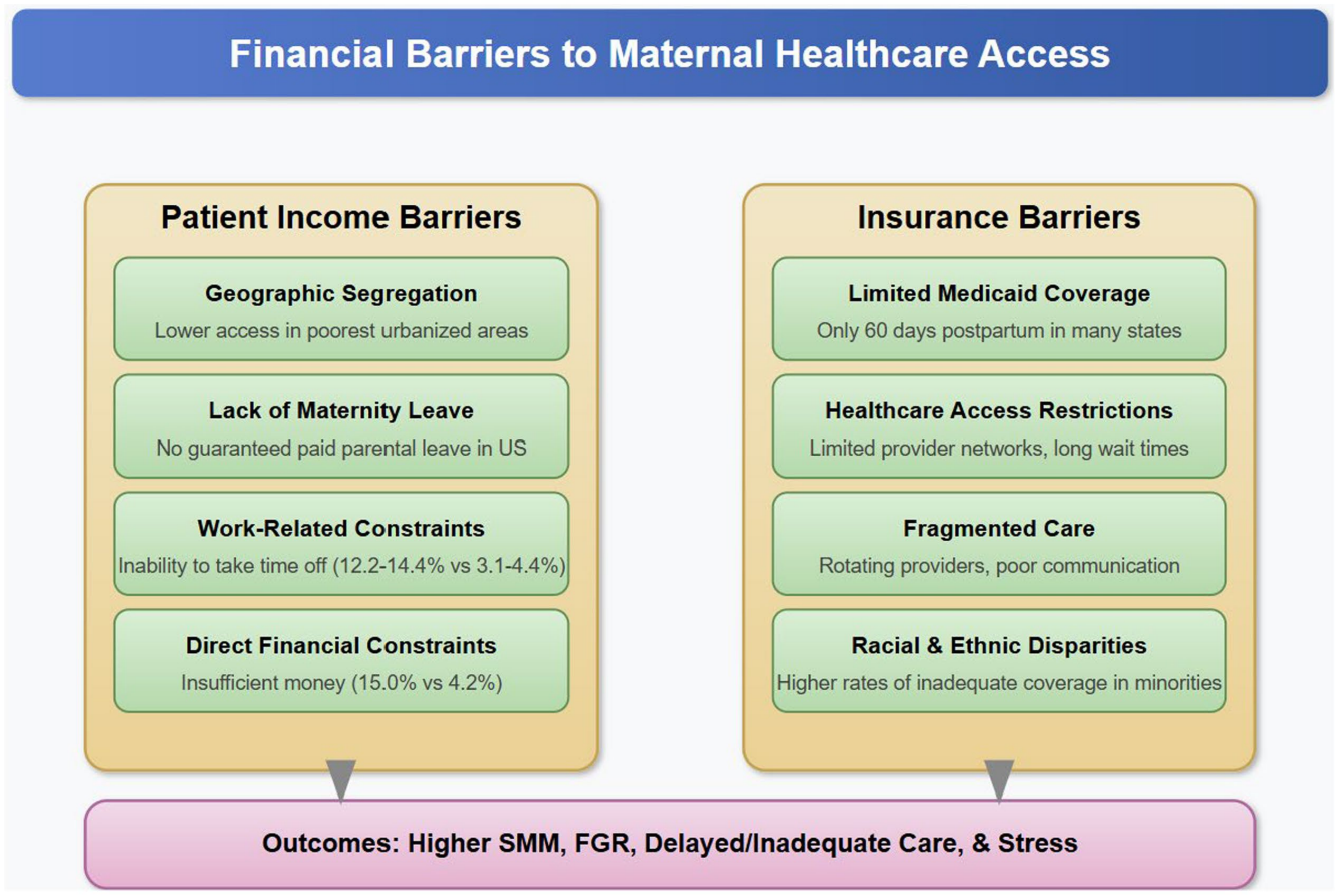
Financial barriers to maternal healthcare access in the United States. This figure illustrates the two primary financial barrier categories (Patient Income and Insurance) and their specific mechanisms that impede equitable maternal healthcare access, particularly for minority and marginalized populations. These barriers contribute to adverse maternal and infant health outcomes including severe maternal morbidity (SMM), fetal growth restriction (FGR), delayed/inadequate care, and increased psychological stress.

Financial barriers to maternal healthcare access primarily reflect income-related constraints and insurance limitations, which disproportionately affect racial and ethnic minority populations (Figure 4).

##### 3.1.1.1 Patient income

Patient income represents a significant financial barrier to accessing maternal healthcare, particularly for minority and marginalized populations. While urban areas may experience inequities in provider distribution related to socioeconomic segregation, maternal healthcare access is most severely constrained in rural settings, where hospital closures, workforce shortages, and long travel distances substantially limit availability and accessibility of care (28). Furthermore, the United States’ inadequate social support infrastructure exacerbates these barriers, ranking at the bottom among developed nations for childcare subsidies and maternity leave benefits as the only developed country without guaranteed paid parental leave (29). Consequently, healthcare providers identify this lack of support as contributing to postpartum depression, with some women returning to work within a week postpartum due to economic necessity (29).

Income disparities demonstrate measurable effects on maternal health outcomes. Women in high-poverty neighborhoods experience substantially higher SMM rates (271.6 vs. 184.8 per 10,000 deliveries) compared to those in low-poverty areas (30). Moreover, the relationship between income and social determinants of health (SDOH) follows an inverse dose–response pattern, with SDOH issues decreasing from 0.5% prevalence in the lowest income quartile to 0.2% in the highest (31). Additionally, those with SDOH issues were 16% more likely to experience fetal growth restriction (FGR) (95% CI 1.04–1.34), with Hispanic women showing 69% increased risk (95% CI 1.25–2.27) (31).

Racial and ethnic disparities in prenatal care access are significantly mediated by income differences. Compared to non-Hispanic White women, Hispanic women were more likely to have delayed prenatal care (AOR 1.84, 95% CI 1.27–2.65) or inadequate prenatal care (AOR 2.01, 95% CI 1.61–2.50), with transportation costs, inability to take unpaid time off work, and geographic distance representing key income-mediated obstacles (32). Notably, income appears to be the largest contributing factor to prenatal care disparities; including income in multivariable models resulted in a 32% decline in the observed odds ratio for delayed prenatal care (32). Hispanic mothers were significantly more likely than White mothers to cite financial constraints (15.0% vs. 4.2%), lack of transportation (16.6% vs. 2.5%), inability to take work leave (12.2% vs. 3.1%), and perceived distance to care (30.6% vs. 9.1%) as barriers, reflecting patient-reported travel burden rather than a fixed mileage threshold (32). The COVID-19 pandemic further intensified financial barriers, with job losses forcing women to prioritize basic bills over mental healthcare (33). Single-parent household prevalence, which is higher among Black families and itself reflects structural inequities including mass incarceration and economic instability, further compounds these financial pressures (33). Thus, financial strain caused by income limitations contributes to elevated stress levels, especially among Black women, highlighting the importance of comprehensive screening for social and structural determinants of health during pregnancy and postpartum periods (33).

##### 3.1.1.2 Insurance

Insurance-related restrictions pose major financial obstacles to accessing maternal healthcare. Prospective patients seeking perinatal mood and anxiety disorder (PMAD) treatment from specialized clinics without prior referrals succeed only approximately 50% of the time, facing waiting periods up to 2 months (34). Furthermore, restrictions on insurance coverage coupled with high self-payment costs create prohibitive out-of-pocket expenses, with lack of concrete information about costs creating additional hurdles for patients struggling to budget for care (34). Although Medicaid covers over four in 10 U.S. births, maternal coverage has significant limitations. In many states, pregnant individuals are only eligible for maternal health coverage through 60 days postpartum—particularly within the 12 states refusing Medicaid expansion under the Affordable Care Act (34). Consequently, this restrictive coverage window limits mothers’ accessibility to various health services, particularly concerning given that at least one-third of maternal deaths occur during the postpartum period beyond this 60-day coverage mark, making Medicaid expansion a critical policy strategy (34).

Racial and ethnic disparities in insurance coverage compound these challenges. African American and Hispanic mothers were more likely than White women to have Medicaid as their primary insurance source or to lack coverage entirely (35). Moreover, compared to non-Hispanic White women, Hispanic women had lower levels of health insurance before pregnancy and higher levels of public insurance and WIC utilization during pregnancy (32). Despite improvements in insurance status, statistically significant disparities in healthcare utilization between Hispanic and non-Hispanic White women persisted in four of five outcome variables: delayed prenatal care, inadequate prenatal care, no 1-week newborn visit, and no well-baby care (32). Additionally, timing of Medicaid activation contributed to these disparities, as Hispanic mothers cited not having sufficient money for prenatal appointments and not having their medical card despite having Medicaid later in pregnancy (32).

Insurance type was associated with maternal health outcomes, with patients in the lowest income quartile and those covered by Medicare having the highest odds of fetal growth restriction (FGR) (31). Patients in urban teaching settings had 34% increased odds of FGR (95% CI 1.27–1.41) than patients admitted in rural hospitals (31). In Pennsylvania Medicaid, researchers found large and statistically significant racial, regional, and MCO disparities in access to prenatal and postpartum care, with worst performance in postpartum care timeliness (36). Furthermore, insurance plan structure affects continuity of care. Lack of continuity during prenatal care was particularly problematic for Black and Latina women, many of whom had rotating prenatal providers owing to their Medicaid insurance plans (37). This hindered trust-building, with lack of familiarity between provider and patient associated with poorer patient-reported peripartum experiences (37). Ultimately, money and insurance coverage were repeatedly mentioned as significant barriers to treatment (33).

#### 3.1.2 Systematic barriers

**Figure 5:**
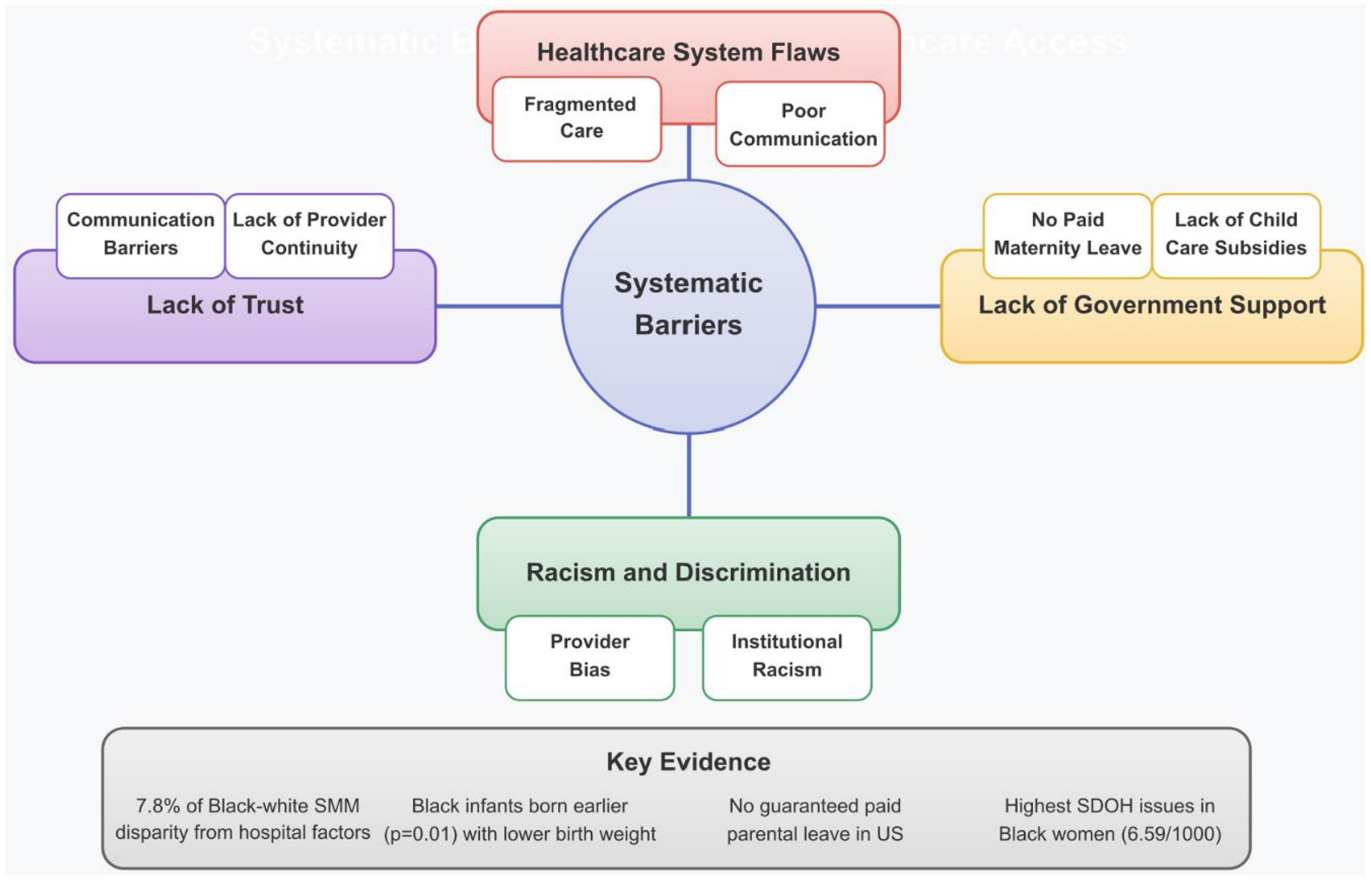
Systematic barriers to maternal healthcare access. The diagram illustrates the four major categories of systematic barriers that impede equitable maternal healthcare access, particularly for minority and marginalized populations. These interconnected barriers operate at structural and institutional levels, affecting maternal care quality and outcomes. “Lack of provider continuity” refers to insurance- and system-driven fragmentation of prenatal and perinatal care documented in Medicaid and safety-net settings, not training-related provider rotation.

Beyond financial constraints, systematic barriers, including healthcare system flaws, limited government support, racism and discrimination, and lack of patient–provider trust, pose major obstacles to equitable maternal healthcare access. As shown in Figure 5, these interconnected barriers operate synergistically, compounding challenges for marginalized populations.

##### 3.1.2.1 Healthcare system flaws

Structural flaws within the maternal healthcare system, including fragmented care and communication gaps, significantly impede equitable care delivery. Women—particularly women of color—reported extended wait times, brief clinical encounters, and inconsistent communication across providers, often resulting in conflicting information and poor care continuity (37, 38).

##### 3.1.2.2 Lack of government support

Governmental policy deficiencies contribute substantially to maternal healthcare disparities. The United States ranks at the bottom among developed nations regarding childcare subsidies and maternity leave benefits, standing as the only developed country without guaranteed paid parental leave (29). Healthcare providers identify this lack of support as a contributing factor to postpartum depression and a complicating factor in delivering adequate care, with some reporting that economic pressures force women to return to work within a week postpartum despite medical recommendations for recovery time (29). Additionally, women in studies frequently mentioned stressors related to these structural deficiencies, including concerns about high maternal mortality rates for Black women, discomfort with hospital birth settings, and self-reported anxiety regarding their children’s future well-being, as described in qualitative interviews and survey responses in the United States (33). These concerns underscore the broader structural issues that require comprehensive policy interventions.

##### 3.1.2.3 Racism and discrimination

Despite greater physician concentration in urbanized areas where many minority women on Medicaid reside, this geographic advantage did not translate to enhanced service access, particularly for non-Hispanic Black women (28). This disconnect illustrates how structural factors—such as differential Medicaid acceptance, provider availability, and institutional practice patterns—that have been shown to produce racially patterned access barriers even in the absence of explicit discriminatory policies (28). Research consistently demonstrates that racial and ethnic differences in SMM persist even after adjustment for covariates. Simulation models revealed that hospital factors accounted for only 7.8% of the Black-White disparity in SMM, suggesting that the majority of racial disparities in maternal morbidity stem from factors beyond hospital quality (39). Specifically, one simulation estimated that 156 fewer SMM cases would have occurred among Black women had they given birth at the same hospitals as White women (39).

Study participants frequently discussed healthcare providers’ role in perpetuating disparities, particularly noting providers’ failure to listen to concerns raised by Black/African American patients. These communication breakdowns often stemmed from implicit biases and stereotypical beliefs, such as the “myth of the strong Black woman,” suggesting Black women can withstand more pain (40). Consequently, many Black/African American participants emphasized the need to identify empathetic medical providers who listen attentively and acknowledge their pain, with some specifically advising pregnant Black women to seek Black doctors to mitigate these issues (40). Moreover, the pervasiveness of institutional racism across service systems, including education, healthcare, housing, justice, and labor significantly influences health trajectories for communities of color, with environmental-related experiences and social status contributing to health disparities with proven transgenerational effects (41).

Statistical evidence further supports racism’s impact on maternal outcomes. *Post hoc* comparisons revealed significant Black/White differences in both birth weight (*p* = 0.002) and gestational age (*p* = 0.01), with infants of Black mothers weighing less and being born earlier despite similar maternal risk profiles (42). Additionally, non-Hispanic Black women had the highest rates of social determinants of health (SDOH) issues (6.59 per 1,000 hospitalizations), followed by Hispanic women (3.82 per 1,000) (31). When compared with non-Hispanic White populations, non-Hispanic Black and non-Hispanic other racial/ethnic groups had 43% and 24% increased odds of fetal growth restriction (FGR), respectively (31). Perinatal outcomes data from quality improvement projects consistently demonstrate significant racial disparities, with Black people experiencing the poorest outcomes across almost all indicators examined (43).

##### 3.1.2.4 Lack of trust

Trust deficits between patients and providers constitute a critical systematic barrier to equitable maternal healthcare, particularly for communities with histories of mistreatment (44). Communication barriers significantly undermine trust, with Hispanic/Latinx patients reporting higher likelihood of clinicians speaking too quickly, using difficult terminology, exhibiting discriminatory behavior (*p* < 0.001), and behaving disrespectfully (*p* = 0.02) compared to White and Black patients (43). Lack of continuity in care further erodes trust, as frequent provider turnover disrupts relationship-building and is associated with poorer peripartum experiences (37). Importantly, interventional evidence indicates that cultural humility training can improve provider awareness, with significant increases in attribution of healthcare disparities to provider behavior (*p* = 0.005) and patient–provider interactions (*p* = 0.02) following implementation (43).

#### 3.1.3 Educational barriers

Educational attainment and neighborhood education levels significantly influence maternal health outcomes. Women residing in higher-education neighborhoods exhibit better outcomes, such as lower viral loads at delivery among pregnant women with HIV (45). Racial and ethnic disparities in education contribute to inequities in healthcare access, as African American and Hispanic mothers are more likely than White mothers to have lower educational attainment, rely on public insurance, and experience delayed or inadequate prenatal care (35). Maternal education has been identified as a major contributor to racial disparities in preterm birth and is significantly associated with SMM after adjustment for sociodemographic factors (30, 46). Educational disadvantage frequently intersects with other social determinants of health, including income and insurance coverage, creating compounded barriers to timely care. Importantly, disparities in prenatal and newborn care utilization between Hispanic and non-Hispanic White women were substantially attenuated after controlling for education, income, and insurance status, underscoring the mediating role of education in maternal healthcare inequities (32).

#### 3.1.4 Geographic barriers

Geographical factors profoundly influence maternal healthcare access, creating substantial disparities through physical distance to facilities, neighborhood characteristics, and regional variations in healthcare resources.

The geographic segregation of lower-income women in impoverished urbanized areas, where physicians rarely establish private practices, severely compromises healthcare access for minority groups (28). Paradoxically, although minority women on Medicaid concentrate in urbanized areas with greater physician density, this proximity fails to translate into enhanced service access, particularly for non-Hispanic Black women (28). Additionally, neighborhood characteristics significantly shape maternal healthcare utilization, with community connections influencing service utilization and attitudes (45). However, neighborhoods with high crime rates create high-stress environments that adversely impact health outcomes through victimization, post-traumatic stress, or depression (45).

Hospital quality and location represent crucial geographical dimensions affecting maternal outcomes. Simulation models demonstrate that if Black women had delivered at the same hospitals as White women, there would be 156 fewer SMM cases, corresponding to a 7.8% reduction in the Black-White SMM disparity (39). Furthermore, physical distance creates formidable barriers: Hispanic women were markedly more likely than White women to perceive doctor’s offices as too distant (30.6% vs. 9.1%) and faced difficulty accessing offices during clinic hours (30.6% vs. 8.3%) (32). In consequence, 54.8% of newborns of Hispanic women received well-baby care at hospital clinics or community health centers, compared to 12.3% of White newborns (32).

Regional variations compound geographic disparities. A Pennsylvania study revealed significant regional differences in prenatal care timeliness (AOR 1.26–1.54), frequency (AOR 1.44–1.80), and postpartum care access across regions (36). Moreover, US-born Hispanic women demonstrated significantly higher social support scores (4.47 ± 0.83) than non-US-born Hispanic women (4.18 ± 0.91) (*p* < 0.001) (47), suggesting geography encompasses both physical location and migratory status, as defined by nativity rather than legal status, which was not disaggregated in the reviewed studies.

#### 3.1.5 Comorbidities

Evidence from pregnant women with HIV highlights the interaction between comorbidities and social determinants of health, as residence in higher-education neighborhoods was associated with lower likelihood of elevated viral loads at delivery, suggesting that supportive community contexts can partially mitigate risks associated with serious pre-existing conditions (45).

### 3.2 What maternal health outcomes and disparities emerge from healthcare access barriers?

**Figure 6:**
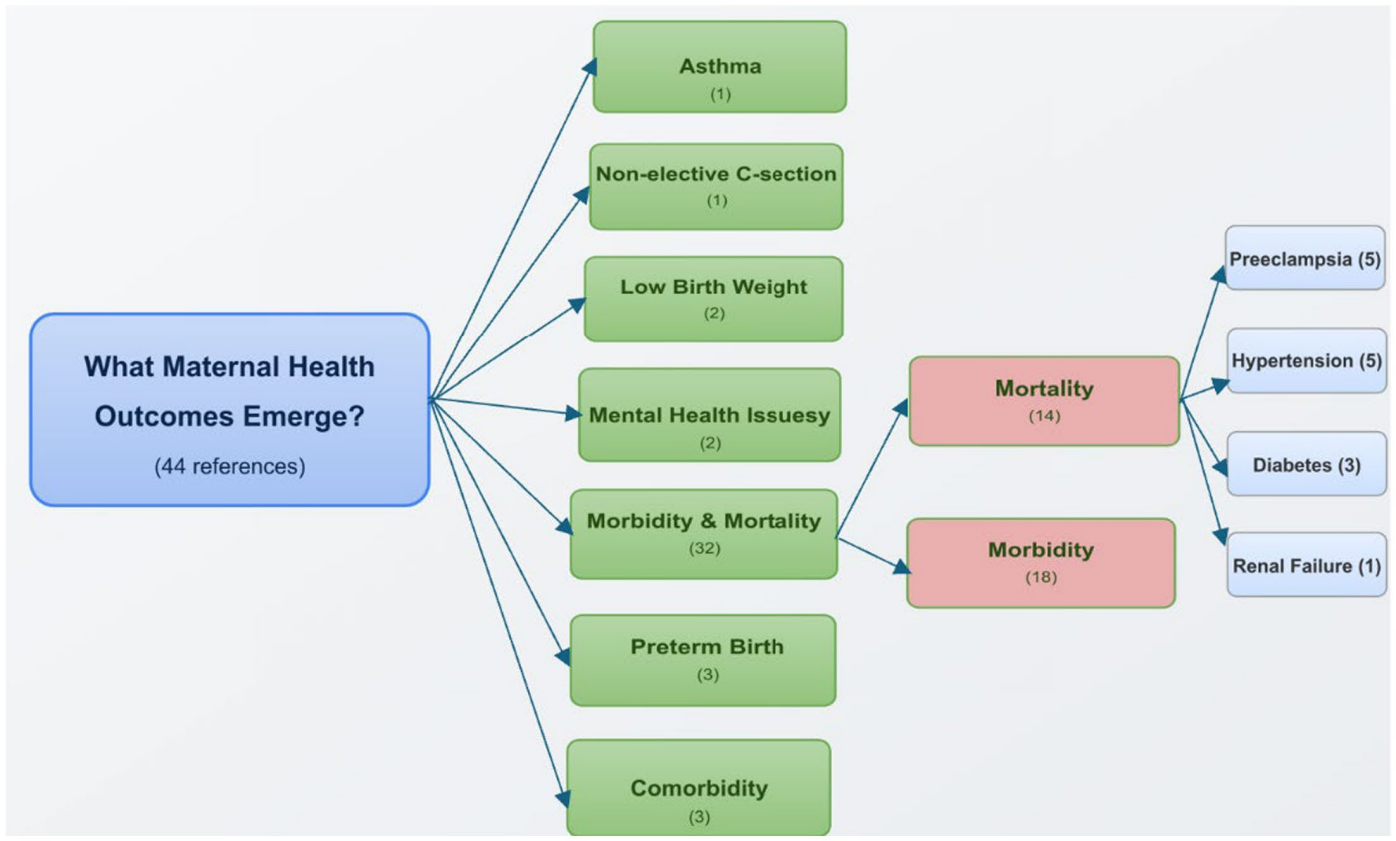
Maternal health outcomes and disparities emerging from healthcare access barriers. The diagram illustrates that morbidity and mortality represent the most frequently cited outcomes, with significant racial disparities in maternal mortality rates. Numbers in parentheses indicate the frequency of references to each outcome category across the systematic review. For analytical purposes, references to different outcome types within the same publication were counted separately to capture the full range of maternal health outcomes discussed in the literature. This approach provides a more comprehensive representation of the various outcomes associated with maternal health disparities.

The systematic literature review identified multiple maternal health outcomes that emerge from barriers to healthcare access, with a total of 44 references addressing these outcomes. As illustrated in Figure 6, these outcomes fall into seven distinct categories: specific health conditions including asthma (1 reference) and comorbidities (3 references); birth-related outcomes including non-elective C-section (1 reference), low birth weight (2 references), and preterm birth (3 references); mental health issues (2 references); and the most frequently cited category of morbidity and mortality (32 references combined).

#### 3.2.1 Specific health conditions

##### 3.2.1.1 Asthma

Asthma during pregnancy represents a health outcome associated with healthcare access barriers. Coded asthma rates during pregnancy were found to be approximately 30% higher in African American women and 40% higher in Hispanics compared to White women, though multiple logistic regression demonstrated that insurance status explained much of this racial disparity (48). This finding highlight how specific health conditions interact with structural barriers such as insurance coverage to create compounded disadvantages for women of color (48).

##### 3.2.1.2 Comorbidities

Comorbidities present a complex relationship with maternal outcomes. Interestingly, in some cases, the presence of pre-existing conditions like cardiovascular disease and diabetes was associated with decreased likelihood of maternal mortality. This counterintuitive finding may be explained by the fact that women with diagnosed chronic conditions often receive more intensive baseline care and monitoring throughout pregnancy (49). For example, a diabetes diagnosis was associated with a 39% decrease in maternal death outcomes among White women and a 34% decrease among African American women (49).

#### 3.2.2 Birth outcomes

##### 3.2.2.1 Non-elective C-section

In US, Non-elective cesarean deliveries showed concerning trends and racial disparities. Cesarean delivery rates increased substantially from 2000 to 2009 for both White women (197.5 to 303.0 per 1,000 deliveries, a 53% increase) and Black women (225.1 to 330.8 per 1,000 deliveries, a 47% increase) (50). Throughout the study period, after adjustment for maternal demographics, parity, and pregnancy characteristics cesarean delivery rates were consistently higher for Black women than for White women (50). However, the extent to which these disparities reflect differences in unmeasured comorbidities versus healthcare quality remains unclear.

##### 3.2.2.2 Low birth weight

Low birth weight (LBW) showed significant racial disparities, with Black women experiencing the highest rates (8.4%), followed by White women and American Indians/Alaska Natives (6.1% each), Asian/Pacific Islanders (5.6%), and Hispanics (4.5%) (51). Very low birth weight (VLBW) followed a similar pattern, with Black mothers experiencing the highest rates (2.4%) (43). Despite experiencing the highest LBW rates, Black mothers were only as likely as White women, and slightly less likely than Asian/Pacific Islanders and Hispanics, to receive postpartum and newborn care (51), highlighting disconnects in the care continuum.

##### 3.2.2.3 Preterm birth

Preterm birth represents another significant adverse outcome. Amphetamine- and opioid-related deliveries were associated with higher preterm delivery levels (51). Geographic, sociodemographic, and maternal health variables accounted for 38 and 31% of excess preterm birth (PTB) and very preterm birth (VPTB) among Black, non-Hispanic women, respectively (46). Furthermore, American Indian/Alaska Native (AI/AN) pregnant people had significantly higher preterm delivery rates (OR 1.52, *p* < 0.0001) and stillborn birth rates (OR 1.30, *p* = 0.0249) compared to non-Hispanic White pregnant people (52).

#### 3.2.3 Mental health issues

Mental health issues emerged as important outcomes related to maternal healthcare barriers, particularly among women who experienced maternal morbidity and disruptions in care. Many women reported emotional and psychological trauma from childbirth, describing their experiences as traumatic, confusing, and stressful, with complicated deliveries often overshadowing positive experiences of new motherhood (37). Several women expressed fear of future deliveries, with some stating they would “never want to have another baby” (37). Equity-related differences were also evident among immigrant populations. Research on foreign-born women found that psychological resilience declined with longer residence in the United States, as depression and stress increased over time, suggesting the cumulative impact of acculturation and systemic barriers on maternal mental health (47).

#### 3.2.4 Morbidity and mortality

The most extensively documented outcomes in the literature pertained to maternal morbidity (18 references) and mortality (14 references), with stark disparities evident along racial and ethnic lines.

##### 3.2.4.1 Morbidity

SMM encompasses 21 distinct delivery complications representing serious, potentially life-threatening conditions requiring intensive medical intervention (52). The prevalence of SMM varied considerably by race and ethnicity. In California from 2007 to 2012, overall SMM prevalence was 1.3%, with the highest rate in Black women (2.1%) and lowest in White women (1.1%) (39). In unadjusted models, Black women had double the SMM risk compared with White women (OR 1.99; 95% CI, 1.92-2.07), while US-born Hispanics (OR 1.25), foreign-born Hispanics (OR 1.24), Asian and Pacific Islanders (OR 1.24), and those categorized as “Other” (OR 1.55) also showed significantly higher risk (39). These disparities persisted even after extensive adjustment, suggesting structural factors contribute substantially to these inequities. Indigenous populations faced particularly high SMM rates. American Indian/Alaska Native (AI/AN) pregnant people experienced SMM at 191 per 10,000 delivery hospitalizations compared to 120 per 10,000 among non-Hispanic White pregnant people (OR 1.60, *p* < 0.0001) (52), with significantly higher rates of blood transfusion (OR 1.83), eclampsia (OR 2.63), pulmonary edema (OR 2.57), and sepsis (OR 2.59) (52). The most common diagnoses for postpartum hospital utilization were general perinatal complications (17.5%), hypertension/eclampsia (12.0%), non-gynecologic infections (10.7%), and wound infections (8.4%) (53).

Substance use during pregnancy was associated with increased morbidity risk, particularly among women experiencing barriers to integrated prenatal and substance use care. SMM risk among mothers with amphetamine use was 1.6 times higher than among those with opioid use (54). Preeclampsia incidence in amphetamine-related deliveries was approximately twice as high as in opioid-related and other hospital deliveries, while preterm birth rates were substantially elevated (amphetamine: 16.7%; opioid: 12.6%; other deliveries: 5.8%) (54). Mental health consequences were pronounced among women experiencing maternal morbidity, with many reporting traumatic childbirth experiences and persistent psychological distress up to 2 years postpartum (37). For some, these experiences overshadowed early motherhood and contributed to fear of future pregnancies or avoidance of subsequent childbirth (37).

##### 3.2.4.2 Mortality

Maternal mortality demonstrates stark racial disparities. Black women experience maternal mortality rates 3 to 4 times higher than White counterparts (34). Among military veterans, the pregnancy-associated mortality ratio (PAMR) was higher for non-White veterans (116.1 per 100,000 live births) compared to White veterans (78.5 per 100,000) (55). In Texas, maternal mortality rates for non-Hispanic Black women more than doubled from 41.6 per 100,000 in 2006–2010 to 85.6 per 100,000 in 2011–2015 (106% increase, *p* < 0.001), remaining more than twice the rate for non-Hispanic White women (56).

Maternal mortality varies significantly by time period and age. Half of all pregnancy-associated deaths among veterans (50%) occurred in the late postpartum period (43-365 days postpartum) (48), with suicide, overdose, and homicide more prevalent in this period, while acute medical events were more common during pregnancy through postpartum day 42 (55). Age emerged as a critical factor: women aged 40 or older had maternal mortality rates of 558.8 per 100,000—27 times the rate for women under 40 (20.7 per 100,000)—and this older age group accounted for 56% of the overall increase in maternal deaths despite representing only 3% of live births (56).

Among veterans, reported causes of maternal death included cardiac arrest or cardiomyopathy, overdose, suicide, homicide, aneurysm, and pneumonia. However, these findings are descriptive in nature, given the small number of cases, and are not intended to represent population-level risk estimates (37). In Texas, maternal mortality rates increased by 92% for direct obstetric causes and 94% for indirect obstetric causes from 2006–2010 to 2011–2015 (56). Direct obstetric causes accounted for 64% and indirect causes 32% of maternal deaths in 2011–2015. Among direct obstetric deaths, significant increases were observed for liver disorders in pregnancy (ICD-10 code O26.6), which increased from 0.7 per 100,000 births in 2006–2010 to 1.7 in 2011–2015 (*p* < 0.01), and for “Other” specified pregnancy-related conditions (O26.8), which increased from 3.8 to 10.4 (*p* < 0.001) (56). Certain pregnancy complications were associated with increased mortality risk. Preterm delivery was linked to increased mortality, with highest risk in individuals with preterm prelabor cesarean delivery (57). Furthermore, preterm deliveries attributable to induced labor were associated with greater mortality risk among Black compared with White participants (57). Hypertensive disorders of pregnancy, particularly superimposed preeclampsia or eclampsia, were associated with increased all-cause mortality risk (57). The 3-fold higher incidence of superimposed preeclampsia or eclampsia in Black compared with White pregnant participants signals that disparities in pregnancy health may have lifelong implications for earlier mortality (57).

### 3.3 What interventions have been effective in reducing maternal health disparities?

**Figure 7:**
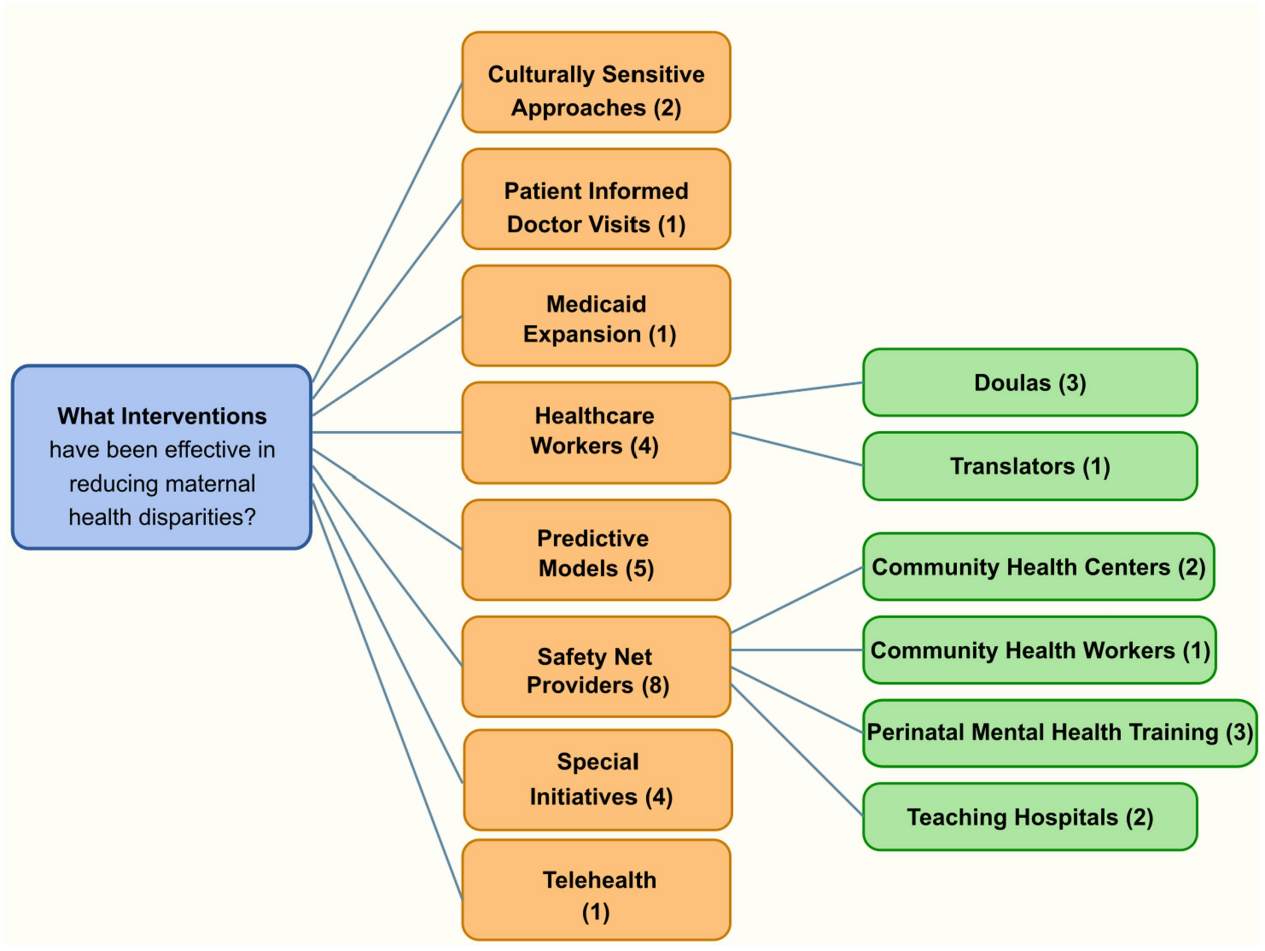
Interventions for reducing maternal health disparities identified in the systematic review. This diagram illustrates the eight major intervention categories and their subcategories, with numbers in parentheses indicating the frequency of references in literature. Safety net providers (8) and predictive models (5) were the most frequently cited intervention types. The frequency counts represent the number of distinct references to each intervention type across the reviewed literature, including multiple references from the same publications when they addressed different intervention categories.

The review identified eight categories of interventions with evidence of effectiveness in reducing maternal health disparities, as summarized in Figure 7. These include culturally sensitive approaches, patient-informed clinical encounters, Medicaid expansion, healthcare workers (e.g., doulas and translators), predictive models, safety net providers, special initiatives, and telehealth. Safety net providers—particularly community health centers, community health workers, perinatal mental health training programs, and teaching hospitals—were most frequently cited, highlighting their central role in addressing access barriers. Collectively, these interventions target structural, financial, and cultural barriers to improve maternal health outcomes among underserved populations.

#### 3.3.1 Culturally sensitive approaches

Culturally sensitive approaches acknowledge and respect patients’ cultural backgrounds, leading to more positive healthcare experiences (48, 58). Two distinct examples highlighted the effectiveness of culturally appropriate interventions. The Native American Women’s Dialog on Infant Mortality (NAWDIN) cradleboard program incorporated traditional counselors into interventions, promoting healing by balancing components of the Indigenous medicine wheel—emotional, physical, spiritual, and mental (58). By examining this case through an Indigenous lens, researchers documented how the program addressed different medicine wheel aspects to achieve balance and health for both mother and infant (58). The MAMA LOVE program addressed leading physical and mental health risk factors that increase maternal morbidity and mortality (53). This intervention added “a layer of screening that contextualizes risk and highlights the importance of evaluating the individual lived experience within the context of ‘risk’” (48). By incorporating mental health and social determinants of health, MAMA LOVE created algorithms that were both usable and effective for end users, making the tool especially valuable for at-risk populations (48). Similarly, community-based doula support for low-income Black and Latina women demonstrated significant outcome improvements: preterm birth rates decreased from 12.4% to 6.3% (*p* < 0.001) and low birthweight from 11.1 to 6.5% (*p* = 0.001) among program participants (59). Thus, these culturally sensitive approaches demonstrate how interventions tailored to specific communities can effectively address unique barriers faced by marginalized populations (48, 59).

#### 3.3.2 Patient-informed doctor visits

Patient-informed doctor visits actively incorporate patient needs and preferences, creating more equitable care experiences (37). Women from White and Asian focus groups described how information and education from providers during prenatal care significantly improved their experiences during emergency deliveries (37). For instance, one participant had previously discussed forceps and vacuum extraction with her provider during prenatal care, which helped her feel prepared and consulted when instrumental delivery became necessary during childbirth (37). This demonstrates how provider-patient communication and shared decision-making can empower women during critical moments, potentially reducing disparities by ensuring all women have the information needed to participate in care decisions (37).

#### 3.3.3 Medicaid expansion

Medicaid expansion represents a significant policy intervention with substantial potential for reducing maternal health disparities (34). Given that Medicaid covers two-thirds of births among Black and American Indian and Alaska Native populations, expanding this program offers “substantial promise for addressing long-standing health inequities” (34). By extending coverage, Medicaid expansion directly addresses financial barriers that disproportionately affect marginalized communities, improving access to critical prenatal, delivery, and postpartum health services, including mental health treatment (34). Furthermore, this policy intervention is particularly important given that financial constraints consistently emerge as major barriers to equitable maternal healthcare access (34).

#### 3.3.4 Healthcare workers

##### 3.3.4.1 Doulas

Doulas have emerged as particularly effective healthcare workers for improving maternal outcomes among underserved populations (58, 59). Studies highlighted their positive impact through community-building among service providers and doulas, creating comprehensive support networks for pregnant women (58). Research discussed the exploration of third-party reimbursement pathways to make doula care financially viable and increase access “in neighborhoods with high poverty, where many pregnant women are not able to pay for these services” (59). However, researchers cautioned that Medicaid offices would need to set rates high enough to provide living wages for doulas to avoid perpetuating systems of inequity (59). The By My Side Birth Support Program demonstrated that “low-income Black and Latina women in Brooklyn neighborhoods are currently underserved by doula support and that when enrolled in these services, they benefit from increased emotional, physical, informational, and social support provided during pregnancy, childbirth, and the postpartum period” (59). Thus, providing free doula support in communities of color and high-poverty neighborhoods may be a critical component in addressing persistent inequities in birth outcomes (59).

##### 3.3.4.2 Translators

Medical translators represent another important category of healthcare workers who can reduce disparities (28). One study found that a greater supply of foreign medical graduates increased the odds of early prenatal care for Hispanics more than for non-Hispanic White women in Florida (28). This suggests that healthcare workers who can bridge language barriers play a crucial role in increasing access to timely maternal care for non-English speaking populations (28). Moreover, by facilitating communication between patients and providers, translators help ensure that all women, regardless of language proficiency, can understand their care options and actively participate in decision-making throughout their maternal healthcare journey (28).

#### 3.3.5 Predictive models

Predictive models have shown significant potential for identifying and addressing maternal health disparities (53, 60). A Composite Postpartum Hospital Utilization (PHU) model found that “because Black and Hispanic people were more likely to have high predicted probabilities,” the model, “coupled with an effective intervention, had the greatest impact on postpartum health inequities” (53). Additionally, “incorporating structural determinants into clinical prediction models may help identify those at risk of poor health due to structural racism and disadvantage” (53). Researchers also developed a Prenatal Care Experiences of People of Color (PCPC) scale to evaluate interventions aimed at improving prenatal care experiences and reducing disparities (53). Furthermore, the RE-AIM framework was utilized to evaluate telehealth intervention implementation in obstetric settings (60). Researchers noted that “given the extensive racial and ethnic disparities in obstetric care, future studies should determine the impact of telehealth specifically on these disparities” (60), emphasizing mechanisms such as removing transportation barriers or increasing self-efficacy through joint decision-making (60). Thus, these predictive models demonstrate the potential for data-driven approaches to identify high-risk populations and tailor interventions accordingly (53, 60).

#### 3.3.6 Safety net providers

##### 3.3.6.1 Community health centers (CHCs)

Community health centers emerged as critical safety net providers for addressing maternal health disparities (51). CHCs provide care for an estimated 3.7% of all births in the U.S., but remarkably 17.2% of all low-socioeconomic status births nationally (51). Furthermore, CHCs account for a larger proportion of births among minorities, caring for approximately 9% of Hispanic births nationally and 21% of all low-SES Black births (51). Despite serving predominantly high-risk populations, CHCs have comparable low birth weight rates to the total U.S. population (7.5% vs. 7.7%) (51). Most significantly, research found that “while research has shown that CHCs may contribute to reducing racial/ethnic disparities in access to care and health status among adults, this is the first study to provide evidence that disparities in some perinatal care and birth outcomes may be lower in CHCs” (51), underscoring the potential of CHCs to serve as equalizing forces in maternal healthcare for underserved populations (51).

##### 3.3.6.2 Community health workers (CHWs)

Community health workers represent another effective safety net strategy for addressing maternal health disparities (44). CHWs “play an important role in facilitating access to prenatal care and coordination of broader maternal and child health care services and community resources, and are particularly effective for improving health outcomes for vulnerable groups or in areas where access to care is an obstacle” (44). Researchers used peer-reviewed literature, qualitative data, and team experiences to frame CHW activities, focusing on establishing clear expectations and roles, providing adequate supervision, incorporating evaluation and feedback regarding reach in communities with greater outcome disparities, and creating opportunities for integrating CHWs into the larger healthcare system through strategic budget models (44). Consequently, this comprehensive approach demonstrates how CHWs can be effectively deployed as frontline workers to address disparities at the community level (44).

##### 3.3.6.3 Community perinatal mental health teams (CPMHTs)

Community Perinatal Mental Health Teams have demonstrated effectiveness in addressing mental health disparities among perinatal women (61). Women with access to CPMHTs had higher numbers of community care contacts (mean 4.31 versus 3.26) compared to those without CPMHT access (61). Additionally, “a lower proportion of women with access to CPMHTs compared to those with no CPMHT had contact with acute care services (admissions and CRTs), a higher proportion had contact with community care services, and, overall, a higher proportion had contact with any secondary mental health services” (61). These results suggest that implementing CPMHTs has supported increased access to secondary mental healthcare services, particularly community mental healthcare (61). Furthermore, following CPMHT implementation, “the percentage of women using acute care decreased while the percentage of women using community care increased” (61), indicating that CPMHTs may help women access care earlier in less intensive settings, potentially preventing crises requiring acute intervention (61).

##### 3.3.6.4 Teaching hospitals

Teaching hospitals serve as important safety net providers, particularly for women insured by Medicaid (28). Research consistently indicates that “the presence or use of these safety net providers was associated with higher odds of prenatal care” (28), with significant evidence of “positive, differential effects of these safety net providers for service receipt by members of minority groups” (28). Furthermore, “a greater supply of foreign medical graduates increased the odds of early prenatal care for Hispanics more than for non-Hispanic White women in Florida” (28), and “the presence of a teaching hospital significantly increased the odds that non-Hispanic black and Hispanic pregnant women received early and adequate prenatal care” (28). Therefore, teaching hospitals play an important role in reducing disparities for minority women who face the greatest barriers to timely and adequate care (28).

#### 3.3.7 Special initiatives

Special initiatives specifically designed to address maternal health disparities have shown promising results (62–64). The enhanced Maternal Care Access Program (eMCAP) “demonstrated improvement in both attendance and quality of postpartum care for women living in underserved areas” (63), with significantly lower HbA1c values and improved chronic hypertension follow-up rates, offering “an opportunity to reduce maternal morbidities and mortalities related to cardiovascular disease—one of the leading causes of pregnancy-related death in the United States” (63).

A Quality Improvement (QI) collaborative focused on SMM showed that the total SMM rate “dropped continuously after the initiation of the QI collaborative” (62), with mean quarterly rates falling from 22.0% in the baseline to 18.6% in the postintervention period (62). Most significantly, “SMM rate was reduced in every racial/ethnic group after the intervention” (62), and “the risk of SMM was no longer greater in Black women compared with White women in the postintervention period after accounting for sociodemographic and clinical factors” (62). The researchers emphasized that “the marked improvement in black rates of SMM from hemorrhage and the narrowing of Black-White difference are important findings suggesting QI efforts can be effective in both improving maternal outcomes and reducing inequities in care delivery for a specific medical condition” (62).

Additionally, the Ohio Maternal Safety Quality Improvement Project (MSQIP) demonstrated that “a robust and comprehensive QI initiative with appropriate support and resources can continue to function and demonstrate resiliency in the setting of a global pandemic” (64), enabling hospitals “to leverage evidence-based guidelines to proactively improve care for and health outcomes of people who experienced a hypertensive event during pregnancy” (64). These special initiatives illustrate how targeted, comprehensive approaches can effectively address specific drivers of maternal health disparities (62–64).

#### 3.3.8 Telehealth

Telehealth has emerged as a promising intervention for improving maternal healthcare access, particularly for underserved populations (60). Research evaluating rapid implementation of telehealth visits for prenatal and postpartum care found it feasible “in a practice serving a low-income, majority Latina/x patient population using a parsimonious set of implementation strategies” (60). Measures of reach and adoption suggested that “uptake of the telehealth intervention was ubiquitous in the practice,” and implementation outcomes indicated “acceptability and appropriateness of telehealth” (60). Furthermore, early assessment suggested that “maintenance of telehealth visits once in-person restrictions were lifted in the state of Massachusetts was achievable and feasible” (60). Thus, telehealth could be a valuable tool for addressing geographic and transportation barriers that disproportionately affect low-income and minority women, potentially reducing disparities in maternal healthcare access and outcomes (60).

## 4 Discussion

### 4.1 Synthesis of key findings

Prior systematic reviews have documented racial and socioeconomic disparities in maternal health outcomes in the United States, with recurring findings related to financial barriers, geographic inaccessibility, and structural racism in healthcare delivery (65–67). The present review corroborates and extends this body of synthesized evidence by integrating barriers, outcomes, and interventions within a single unified framework, and by applying an explicit equity lens across all stages of the review process. Notably, the present findings align with prior reviews identifying Black women as disproportionately affected by severe maternal morbidity and mortality, while adding updated evidence on Indigenous populations and the growing role of telehealth and predictive modeling as equity-oriented interventions.

Financial barriers emerged as significant obstacles, encompassing patient income limitations and insurance restrictions (29–37). Hispanic mothers were significantly more likely than White mothers to cite financial constraints (15.0% vs. 4.2%) as obstacles to accessing prenatal care (31). The relationship between income and social determinants of health followed an inverse dose–response pattern, with SDOH issues decreasing as income levels rose (33). Systematic barriers, including healthcare system flaws, lack of government support, racism and discrimination, and lack of trust, represented pervasive institutional obstacles disproportionately affecting minority populations. Simulation models revealed that hospital factors accounted for only 7.8% of the Black-White disparity in severe maternal morbidity (SMM), suggesting systemic racism operates through multiple channels beyond hospital quality (39, 40). Communication breakdowns often stemmed from implicit biases such as the “myth of the strong Black woman” (41), highlighting the need for comprehensive policy and institutional changes.

Educational and geographical factors further compounded these challenges. Physical distance to healthcare facilities created formidable barriers, with Hispanic women markedly more likely than White women to report that doctor’s offices were too distant (30.6% vs. 9.1%) (32). Regional variations in care quality underscore the determinative role of geographic location in maternal healthcare access (37).

The review identified concerning disparities in maternal health outcomes across racial and ethnic lines. Black women experience maternal mortality rates 3 to 4 times higher than White counterparts (35), with overall SMM prevalence of 1.3%—highest in Black women (2.1%) and lowest in White women (1.1%) (40). Indigenous populations faced particularly high SMM rates, with American Indian/Alaska Native pregnant people experiencing SMM at 191 per 10,000 delivery hospitalizations compared to 120 per 10,000 among non-Hispanic White pregnant people (53).

Several promising interventions were identified, with safety net providers and predictive models most frequently cited. Culturally sensitive approaches incorporated traditional elements and addressed social determinants of health (33, 59), while patient-informed care prioritized communication and shared decision-making (58). Healthcare workers, including doulas (59, 60) and translators (29), improved access and quality of maternal care. Safety net providers, including Community Health Centers (46), Community Health Workers (38), Community Perinatal Mental Health Teams (63), and teaching hospitals (22), expanded access for vulnerable populations. Furthermore, special initiatives and quality improvement projects (62–64) demonstrated that targeted approaches can effectively address specific drivers of maternal health disparities, with one study showing that QI efforts reduced SMM rates in every racial/ethnic group (64). Additionally, policy interventions such as Medicaid expansion (29) and telehealth implementation (61) showed promise in addressing financial and geographical barriers.

### 4.2 Implications for policy and practice

The following implications are directly grounded in findings from the 44 included studies. Evidence from multiple studies consistently demonstrated that financial barriers, including insurance gaps and income limitations, were among the strongest predictors of reduced prenatal care utilization and adverse outcomes (31, 32, 36). Culturally sensitive care models and community health worker integration were associated with measurable improvements in birth outcomes across racially diverse populations (46, 58). Quality improvement initiatives targeting specific clinical drivers of disparity demonstrated the capacity to reduce severe maternal morbidity rates across racial and ethnic groups (62, 64).

Building on this evidence base, the following practice and policy recommendations are proposed. Medicaid coverage should be extended beyond the current 60-day postpartum limit to address the documented gap in postpartum care access (29). Healthcare systems should institutionalize culturally sensitive care frameworks and integrate diverse healthcare workers, including doulas and community health workers, into routine maternal care teams (59). Telehealth policies should be developed with equity considerations to ensure broadband access does not further disadvantage rural and low-income populations (27). Systemic racism within healthcare institutions should be addressed through mandatory implicit bias training, transparent disparity reporting, and accountability mechanisms at the institutional level (40).

### 4.3 Strengths and limitations

Strengths of this review include its comprehensive search strategy spanning three decades, mixed-methods synthesis approach integrating quantitative and qualitative evidence, application of equity lenses throughout the review process, rigorous screening following PRISMA guidelines, and structured organization around three central research questions. Limitations include the focus on English-language literature from the United States, potential publication bias, limited depth on specific topics due to the broad scope, heterogeneity of included studies making direct comparisons challenging, and the absence of studies examining Medicaid reimbursement rate impacts on provider participation or maternal health outcomes, representing an important policy-relevant evidence gap. Additionally, the approach of counting references to different aspects within the same publications separately influenced frequency counts for various themes. While our stringent inclusion criteria resulted in 44 studies for synthesis, this focused selection prioritized studies providing integrated evidence across barriers, outcomes, and interventions.

Several methodological gaps in the existing literature warrant attention in future research. The majority of included studies relied on cross-sectional or retrospective designs, limiting causal inference regarding the relationship between barriers and outcomes. Intervention studies with rigorous experimental or quasi-experimental designs remain scarce, making it difficult to establish the comparative effectiveness of different approaches. Indigenous women, immigrant populations, and rural residents were underrepresented across included studies, and future reviews should prioritize targeted searches for evidence from these groups. Standardized outcome measurement across maternal health disparity research is also needed to enable future meta-analytic synthesis. Longitudinal studies examining the sustained effects of policy interventions, including Medicaid expansion and telehealth implementation, on maternal health equity represent a particularly important and underdeveloped area of inquiry.

## 5 Conclusion

This systematic literature review provides comprehensive analysis of the maternal health crisis in the United States, examining barriers to healthcare access, health outcomes, and effective interventions. The findings reveal complex interplay of financial, systematic, educational, geographical, and comorbidity-related barriers disproportionately affecting minority and marginalized populations. Black and Indigenous women face significantly higher risks of mortality and morbidity compared to White counterparts, with disparities persisting even after controlling for socioeconomic factors, pointing to deeply embedded systemic inequities.

Promising interventions include safety net providers such as Community Health Centers and Community Perinatal Mental Health Teams, culturally sensitive approaches incorporating traditional elements, healthcare workers including doulas and translators, and quality improvement initiatives demonstrating ability to reduce severe maternal morbidity across all racial and ethnic groups. Addressing this crisis requires coordinated action at multiple levels. Critical policy recommendations include expanding Medicaid coverage beyond the 60-day postpartum limit, implementing comprehensive approaches addressing social determinants of health, supporting integration of diverse healthcare workers, and developing telehealth policies expanding access. Healthcare systems should prioritize culturally sensitive, patient-informed care while actively addressing systematic barriers, particularly racism and discrimination. Future research should evaluate long-term impacts of combined interventions, examine cost-effectiveness, and develop implementation strategies for scaling successful programs, with particular attention to unique needs of Indigenous women, immigrants, and rural residents.

## Data Availability

The raw data supporting the conclusions of this article will be made available by the authors, without undue reservation.
