# Supplementary material for: Addressing the U.S. maternal health crisis: a systematic review of healthcare access barriers, disparate outcomes, and effective interventions
Source: Front Public Health. 2026 Apr 23;14:1814063. doi: 10.3389/fpubh.2026.1814063 (PMC13149117; doi:10.3389/fpubh.2026.1814063)
Supplement: Supplementary file 1 [file Table_1.DOCX]

**Supplementary Table 1.** *Characteristics of Included Studies (N = 44).* *Note.* RQ = Research Question; SMM = Severe Maternal Morbidity; SDOH = Social Determinants of Health; AI/AN = American Indian/Alaska Native; QI = Quality Improvement.

| **Author(s) & Year** | **Study Design** | **Sample Characteristics** | **Key Outcomes** | **RQ** | **Primary Findings** |
| --- | --- | --- | --- | --- | --- |
| Adams et al. (2005) | Cross-sectional | Medicaid-enrolled pregnant women; national US data | Prenatal care access, insurance coverage | RQ1, RQ2 | Black and Hispanic women on Medicaid experienced lower access to prenatal care compared to White counterparts; geographic and racial variation in Medicaid access was documented. |
| Admon et al. (2019) | Retrospective cohort | US hospital discharge data 2004–2015; amphetamine/opioid-affected births | Birth incidence, maternal/neonatal outcomes, costs | RQ2 | Amphetamine- and opioid-affected births increased substantially; associated with higher preterm delivery, low birth weight, and significant healthcare costs. |
| Altman et al. (2020) | Qualitative | Women of color with recent birth experience; California | Patient experiences, care recommendations | RQ1, RQ3 | Women of color identified systemic racism, lack of cultural humility, and poor provider communication as barriers; recommended respectful, culturally responsive care. |
| Amore et al. (2023) | Mixed methods (development & evaluation) | Pregnant/postpartum women; web-based program (MAMA LOVE) | Maternal morbidity/mortality risk factors, engagement | RQ3 | Web-based intervention targeting risk factors showed potential for reducing disparities; user engagement was higher among Black and Hispanic participants. |
| Arrington et al. (2021) | Quality improvement project | Obstetric patients; peripartum disparity reduction bundle | Peripartum racial/ethnic disparities | RQ3 | Implementation of a disparity-reduction bundle improved equity metrics; highlighted feasibility of structured QI approaches in addressing racial disparities in peripartum care. |
| Baumgartel et al. (2023) | Cross-sectional | Pregnant Hispanic women; southeastern US | SDOH screening outcomes, psychological outcomes | RQ1, RQ2 | Hispanic pregnant women reported elevated SDOH burden; food insecurity and housing instability were associated with adverse psychological outcomes including depression and anxiety. |
| Bromley et al. (2012) | Cross-sectional | Hispanic and non-Hispanic White women; Rhode Island Medicaid | Prenatal care utilization, insurance barriers | RQ1 | Hispanic women experienced delayed prenatal care initiation and fewer visits; income and language barriers were primary drivers of disparities in healthcare utilization. |
| Chen et al. (2023) | Retrospective analysis | US national vital statistics 2018–2020 | Pregnancy-related mortality rates by race | RQ2 | Pregnancy-related mortality increased during the study period; Black women experienced rates 2–3 times higher than White women across all cause categories. |
| Chung et al. (2004) | Cross-sectional | Pregnant women with asthma; New Jersey vital statistics | Prenatal care utilization, insurance type | RQ1, RQ2 | Race, insurance status, and education were significant predictors of prenatal care utilization among pregnant women with asthma; uninsured women had the lowest utilization. |
| Collier & Molina (2019) | Narrative review | National US data; review of literature | Maternal mortality trends, causes, solutions | RQ2, RQ3 | Maternal mortality in the US has risen while peer nations declined; cardiovascular disease and mental health conditions are leading causes; structural racism compounds disparities. |
| Combellick et al. (2024) | Retrospective cohort | Veterans Health Administration patients; non-obstetric settings | Maternal mortality surveillance, safety net adequacy | RQ2, RQ3 | Significant gaps in maternal mortality surveillance were identified in non-obstetric settings; recommended expanded protocols for non-obstetric providers to improve safety net. |
| Dembosky (2021) | Descriptive/case study | Postpartum women in psychiatric crisis; US health systems | Access to perinatal psychiatric care | RQ1, RQ3 | Postpartum psychiatric care remains severely limited; integrated models co-locating obstetric and mental health services improved access for high-risk postpartum women. |
| Dongarwar et al. (2023) | Retrospective cohort | Racially/ethnically diverse pregnant women; national US data | Fetal growth restriction, SDOH | RQ1, RQ2 | SDOH characteristics including poverty and low education significantly increased fetal growth restriction risk; disparities were most pronounced among Black and Native American women. |
| Gilliam et al. (2024) | Qualitative review | Black maternal health literature and clinical data | Mental health, financial barriers, intersectionality | RQ1, RQ2 | Intersectional stressors including financial strain, systemic racism, and mental health burden uniquely shape Black women's maternal health experiences; policy responses must be intersectional. |
| Glazer & Howell (2021) | Narrative review | US maternal mortality data and mental health literature | Maternal mortality, mental health disparities | RQ2, RQ3 | Mental health conditions are significantly underrecognized contributors to maternal mortality; integrated behavioral health in obstetric care is an evidence-supported pathway forward. |
| Hinkle et al. (2023) | Prospective cohort | Diverse pregnant women; longitudinal follow-up | Long-term mortality following pregnancy complications | RQ2 | Pregnancy complications including hypertension and diabetes were associated with significantly elevated long-term mortality risk; disparities were greater for Black and Hispanic women. |
| Howell et al. (2013) | Retrospective cohort | Obstetric patients; New York City hospitals | Obstetric quality indicators, maternal/neonatal mortality | RQ2 | Paradoxical trends observed: overall obstetric quality improved while racial disparities in maternal and neonatal mortality persisted, driven by hospital-level segregation. |
| Howland et al. (2019) | Retrospective cohort | Delivery hospitalizations; New York City 2008–2012 | Severe maternal morbidity (SMM), racial disparities | RQ2 | Black women had significantly higher SMM rates; neighborhood socioeconomic disadvantage and hospital quality independently contributed to racial disparities in SMM. |
| Inyang (2020) | Quantitative (dissertation) | National death certificate data; African American women | Maternal mortality, chronic disease burden | RQ2 | Chronic disease burden including diabetes and cardiovascular disease was significantly associated with maternal mortality among African American women; disparities persisted after controlling for comorbidities. |
| James et al. (2021) | Mixed methods | American Indian and Alaska Native pregnant/perinatal women | SDOH screening, cultural intervention effectiveness | RQ1, RQ3 | Culturally grounded prenatal interventions addressing SDOH improved engagement among AI/AN women; trust-building and traditional practices were identified as key facilitating factors. |
| Janevic et al. (2024) | Retrospective cohort | Postpartum women; equity-focused prediction modeling | Postpartum hospital utilization, prediction accuracy | RQ2, RQ3 | An equity-focused prediction model identified high-risk postpartum patients with improved accuracy for racial/ethnic minorities; demonstrated feasibility of equity-centered predictive analytics. |
| Kim et al. (2024) | Narrative review | US maternal mortality literature | Epidemiologic trends, disparities, clinical frameworks | RQ2 | Maternal mortality trends reflect growing cardiovascular risk and persistent racial disparities; proposed anesthesiological frameworks for improving obstetric safety across diverse populations. |
| Reisinger-Kindle et al. (2021) | Mixed methods (evaluation) | Prenatal/postpartum patients; academic clinic Massachusetts | Telehealth implementation, access equity | RQ3 | Rapid telehealth implementation during COVID-19 maintained access for most patients; disparities emerged for patients with limited broadband access, primarily low-income and rural populations. |
| MacDorman et al. (2018) | Retrospective analysis | Texas maternal deaths 2006–2015 | Maternal mortality by age, race, cause | RQ2 | Maternal mortality rates in Texas increased substantially; Black women experienced the highest rates across all age groups; cardiovascular conditions and hemorrhage were leading causes. |
| MacDorman et al. (2021) | Retrospective analysis | US national maternal mortality data 2016–2017 | Excess mortality risk for women aged 35+ | RQ2 | Women aged 35 and older faced disproportionately elevated mortality risk from hemorrhage and cardiomyopathy; age-race interactions compounded mortality risk for older Black women. |
| Main et al. (2020) | Quality improvement collaborative | California hospital network; multiracial obstetric patients | SMM from hemorrhage, racial disparities | RQ3 | A large-scale QI collaborative significantly reduced SMM from hemorrhage across all racial/ethnic groups; the initiative narrowed but did not eliminate racial disparities in SMM rates. |
| Momplaisir et al. (2020) | Retrospective cohort | Pregnant women with HIV; Philadelphia | HIV viral load at delivery, neighborhood exposures | RQ1, RQ2 | Adverse neighborhood exposures including poverty and crime were significantly associated with detectable HIV viral load at delivery; racial residential segregation mediated these associations. |
| Morain et al. (2023) | Qualitative | Patients and providers; perinatal mental health systems | System-level barriers to perinatal mental health care | RQ1 | System-level barriers including insurance gaps, provider shortages, and stigma severely limited perinatal mental health access; low-income and minority women were disproportionately affected. |
| Mujahid et al. (2021) | Retrospective cohort | California delivery hospitalizations; statewide data | SMM by birth hospital and race | RQ2, RQ3 | Birth hospital quality explained a significant portion of racial SMM disparities; Black women were disproportionately concentrated in lower-quality hospitals, compounding outcome disparities. |
| Nelson et al. (2018) | Retrospective analysis | US national maternal mortality data 1997–2012 | Population-level factors associated with maternal mortality | RQ2 | Socioeconomic factors including poverty rate and uninsurance were significantly associated with state-level maternal mortality; rural and minority-majority states showed highest rates. |
| Nelson et al. (2023) | Quality improvement project | Postpartum patients; Texas hospital system | Postpartum care utilization, disparity reduction | RQ3 | Extended postpartum care initiative improved follow-up visit rates and chronic disease management; disparities in postpartum care attendance were reduced among low-income and minority patients. |
| Obeng et al. (2023) | Qualitative | Black women with experience of maternal health system; US | Experiences of racism, mortality perspectives | RQ1, RQ2 | Black women described pervasive discrimination, dismissal of pain, and implicit bias as systemic barriers; structural racism was identified as the primary driver of mortality disparities. |
| Parekh et al. (2018) | Retrospective cohort | Medicaid-enrolled pregnant/postpartum women; Pennsylvania | Prenatal/postpartum care disparities | RQ1, RQ2 | Racial and ethnic disparities in prenatal and postpartum care utilization persisted within Medicaid; Black and Hispanic women had lower postpartum visit rates despite insurance coverage. |
| Patel et al. (2021) | Participatory/mixed methods | Pregnant women; Texas community-academic partnership | Pregnancy outcomes, community engagement | RQ3 | A state-community-academic model improved pregnancy outcomes in underserved communities; community health worker integration and trust-building were identified as critical success factors. |
| Roese et al. (2024) | Retrospective cohort (multilevel logistic) | American Indian/Alaska Native pregnant people; Pacific Northwest | Severe maternal morbidity, community-level predictors | RQ1, RQ2 | Community-level factors including poverty and rurality significantly predicted SMM; AI/AN women had substantially higher SMM and preterm birth rates compared to non-Hispanic White women. |
| Schneider et al. (2023) | Quality improvement collaborative | Ohio hospital network; perinatal hypertension patients | Hypertension management, maternal safety outcomes | RQ3 | Statewide perinatal hypertension QI initiative during COVID-19 demonstrated feasibility and improved protocol adherence; early results showed reductions in severe hypertension-related complications. |
| Shi et al. (2004) | Retrospective cohort | Community health center patients; national US data | Perinatal disparities, birth outcomes | RQ2, RQ3 | Community health centers significantly reduced racial/ethnic perinatal disparities including preterm birth and low birth weight; access to safety net providers was protective for minority populations. |
| Tassie et al. (2024) | Retrospective cohort (national) | Pregnant women with specialist mental health history; England | Service use patterns, community mental health team impact | RQ3 | Community perinatal mental health teams increased service access and reduced acute psychiatric admissions; women with histories of specialist care showed improved community engagement post-implementation. |
| Thoma et al. (2019a/b) | Retrospective analysis | US national birth data; Black and White women | Preterm birth disparities, geographic/social determinants | RQ1, RQ2 | Geographic, social, and health determinants accounted for 38% of excess preterm birth among Black women; neighborhood poverty and segregation were the strongest modifiable determinants. |
| Thomas et al. (2014) | Retrospective cohort | High-risk prenatal clinic patients; diverse population | Birth outcome disparities, maternal characteristics | RQ1, RQ2 | Maternal sociodemographic differences explained only part of birth outcome disparities within a high-risk clinic; structural factors independent of individual risk profiles drove remaining inequities. |
| Thomas et al. (2017) | Program evaluation/mixed methods | Underserved pregnant women; Healthy Start doula program | Preterm birth, low birth weight, doula access | RQ3 | Doula services within Healthy Start reduced preterm birth from 12.4% to 6.3% and low birth weight from 11.1% to 6.5%; culturally concordant doula support was identified as a key mechanism. |
| Wang et al. (2021) | Qualitative | Women of diverse racial/ethnic backgrounds with SMM experience | Peripartum care experiences, racial disparities | RQ1, RQ2 | Women of color described dismissive care, pain minimization, and lack of informed consent; insurance status and race interacted to shape the quality of peripartum care received. |
| Wang S. et al. (2023) | Narrative review | US national maternal mortality literature | Maternal mortality trends, cardiovascular risk, prevention | RQ2, RQ3 | Cardiovascular disease is now the leading cause of maternal mortality; prevention opportunities include pre-pregnancy optimization, improved postpartum surveillance, and addressing SDOH. |
| Willis et al. (2014) | Narrative review | US perinatal outcomes literature | Racial disparities in perinatal outcomes, interventions | RQ2, RQ3 | Racial disparities in perinatal outcomes are attributable to multilevel structural factors; evidence-based interventions must address provider bias, systemic racism, and socioeconomic inequities simultaneously. |

*Note. RQ = Research Question; SMM = Severe Maternal Morbidity; SDOH = Social Determinants of Health; AI/AN = American Indian/Alaska Native; QI = Quality Improvement.*
